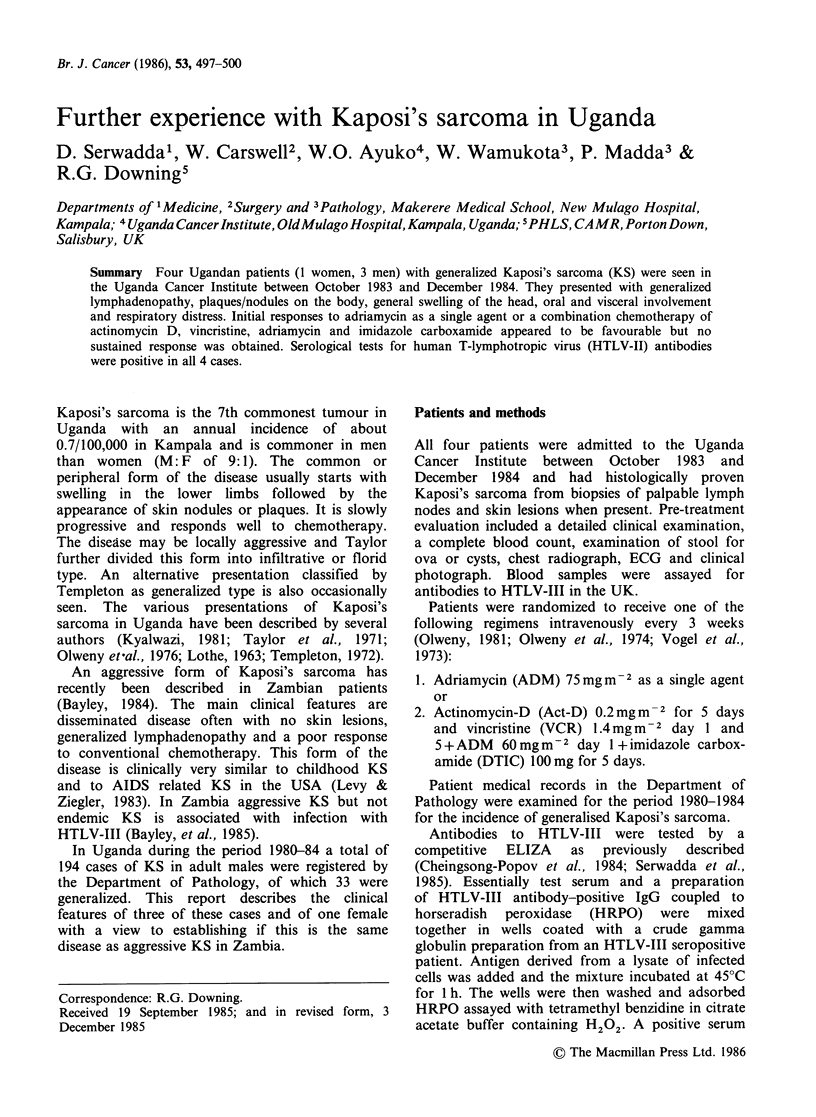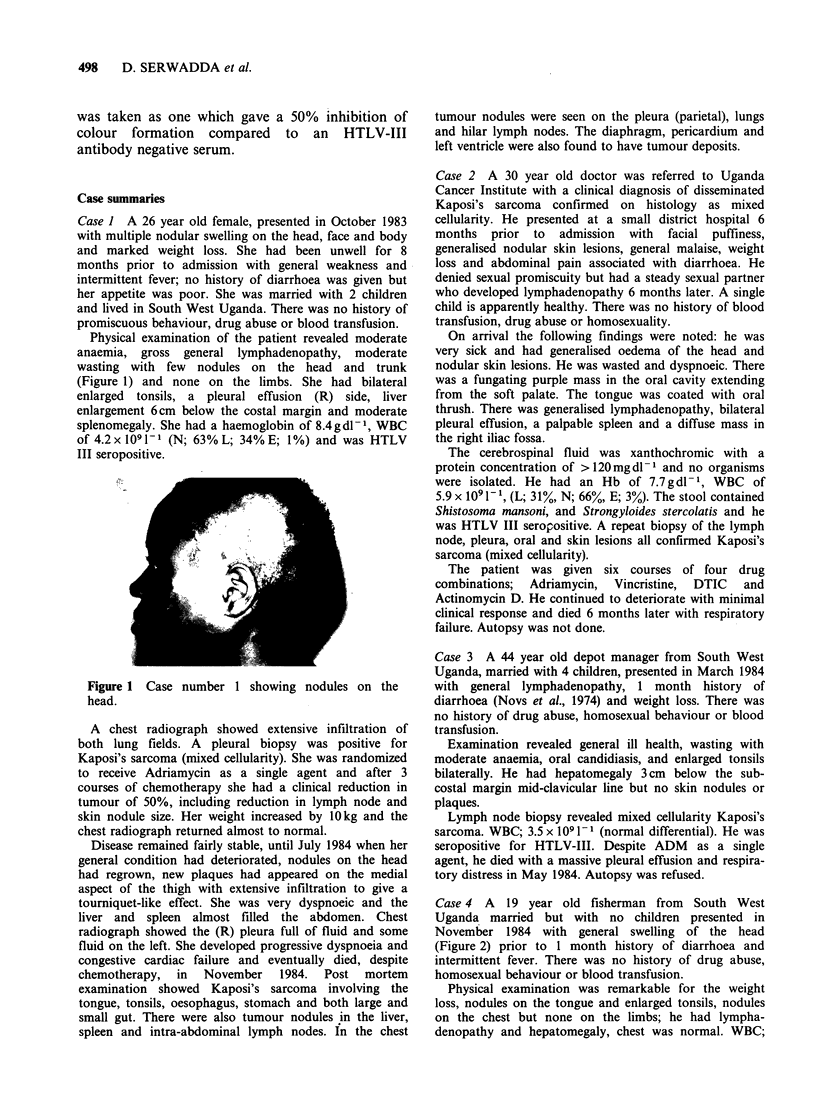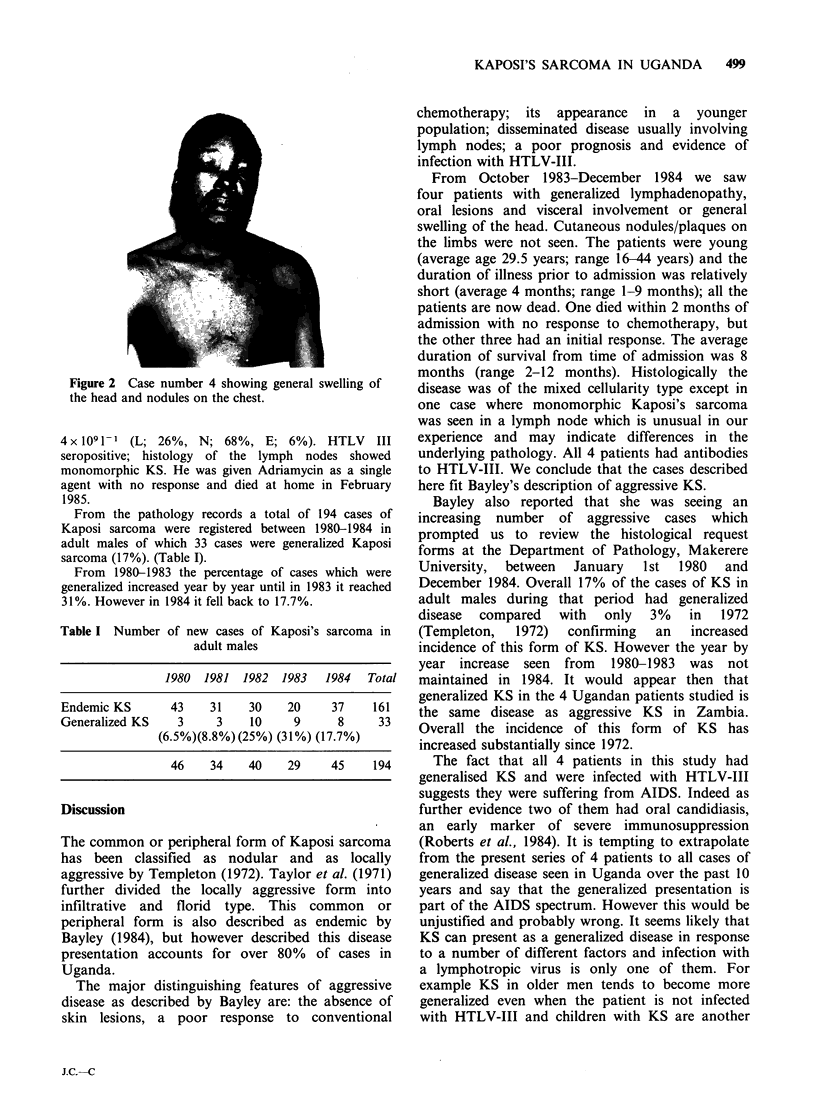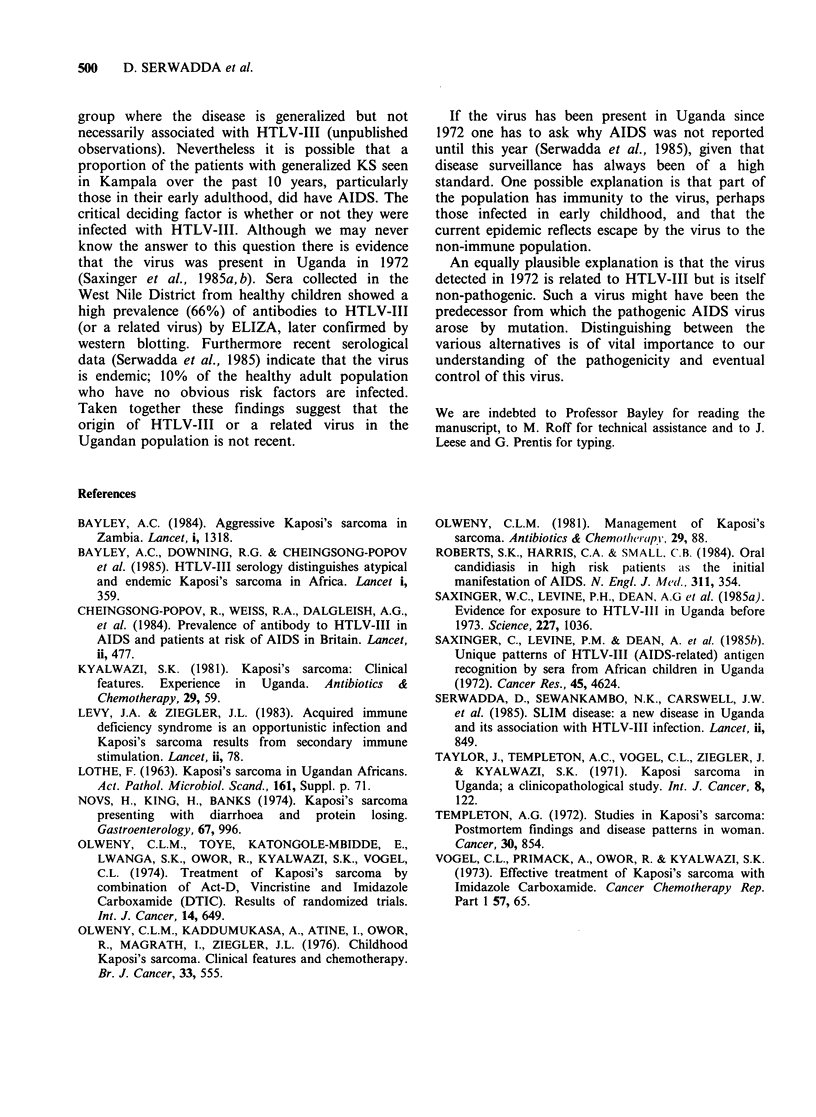# Further experience with Kaposi's sarcoma in Uganda.

**DOI:** 10.1038/bjc.1986.78

**Published:** 1986-04

**Authors:** D. Serwadda, W. Carswell, W. O. Ayuko, W. Wamukota, P. Madda, R. G. Downing

## Abstract

**Images:**


Br. J. Cancer (1986), 53, 497–500

### Further experience with Kaposi's sarcoma in Uganda

D. Serwadda[1], W. Carswell[2], W.O. Ayuko[4], W. Wamukota[3], P. Madda[3] & R.G. Downing[5]

*Departments of [1]Medicine, [2]Surgery and [3]Pathology, Makerere Medical School, New Mulago Hospital, Kampala; [4]Uganda Cancer Institute, Old Mulago Hospital, Kampala, Uganda; [5]PHLS, CAMR, Porton Down, Salisbury, UK*

**Summary** Four Ugandan patients (1 women, 3 men) with generalized Kaposi's sarcoma (KS) were seen in the Uganda Cancer Institute between October 1983 and December 1984. They presented with generalized lymphadenopathy, plaques/nodules on the body, general swelling of the head, oral and visceral involvement and respiratory distress. Initial responses to adriamycin as a single agent or a combination chemotherapy of actinomycin D, vincristine, adriamycin and imidazole carboxamide appeared to be favourable but no sustained response was obtained. Serological tests for human T-lymphotropic virus (HTLV-II) antibodies were positive in all 4 cases.

Kaposi's sarcoma is the 7th commonest tumour in Uganda with an annual incidence of about 0.7/100,000 in Kampala and is commoner in men than women (M:F of 9:1). The common or peripheral form of the disease usually starts with swelling in the lower limbs followed by the appearance of skin nodules or plaques. It is slowly progressive and responds well to chemotherapy. The disease may be locally aggressive and Taylor further divided this form into infiltrative or florid type. An alternative presentation classified by Templeton as generalized type is also occasionally seen. The various presentations of Kaposi's sarcoma in Uganda have been described by several authors (Kyalwazi, 1981; Taylor *et al.*, 1971; Olweny *et al.*, 1976; Lothe, 1963; Templeton, 1972).

An aggressive form of Kaposi's sarcoma has recently been described in Zambian patients (Bayley, 1984). The main clinical features are disseminated disease often with no skin lesions, generalized lymphadenopathy and a poor response to conventional chemotherapy. This form of the disease is clinically very similar to childhood KS and to AIDS related KS in the USA (Levy & Ziegler, 1983). In Zambia aggressive KS but not endemic KS is associated with infection with HTLV-III (Bayley, *et al.*, 1985).

In Uganda during the period 1980–84 a total of 194 cases of KS in adult males were registered by the Department of Pathology, of which 33 were generalized. This report describes the clinical features of three of these cases and of one female with a view to establishing if this is the same disease as aggressive KS in Zambia.

Correspondence: R.G. Downing.
Received 19 September 1985; and in revised form, 3 December 1985

### Patients and methods

All four patients were admitted to the Uganda Cancer Institute between October 1983 and December 1984 and had histologically proven Kaposi's sarcoma from biopsies of palpable lymph nodes and skin lesions when present. Pre-treatment evaluation included a detailed clinical examination, a complete blood count, examination of stool for ova or cysts, chest radiograph, ECG and clinical photograph. Blood samples were assayed for antibodies to HTLV-III in the UK.

Patients were randomized to receive one of the following regimens intravenously every 3 weeks (Olweny, 1981; Olweny *et al.*, 1974; Vogel *et al.*, 1973):

1. Adriamycin (ADM) 75 mg m$^{-2}$ as a single agent or
2. Actinomycin-D (Act-D) 0.2 mg m$^{-2}$ for 5 days and vincristine (VCR) 1.4 mg m$^{-2}$ day 1 and 5+ADM 60 mg m$^{-2}$ day 1+imidazole carboxamide (DTIC) 100 mg for 5 days.

Patient medical records in the Department of Pathology were examined for the period 1980–1984 for the incidence of generalised Kaposi's sarcoma.

Antibodies to HTLV-III were tested by a competitive ELIZA as previously described (Cheingsong-Popov *et al.*, 1984; Serwadda *et al.*, 1985). Essentially test serum and a preparation of HTLV-III antibody–positive IgG coupled to horseradish peroxidase (HRPO) were mixed together in wells coated with a crude gamma globulin preparation from an HTLV-III seropositive patient. Antigen derived from a lysate of infected cells was added and the mixture incubated at 45°C for 1 h. The wells were then washed and adsorbed HRPO assayed with tetramethyl benzidine in citrate acetate buffer containing $H_2O_2$. A positive serum

© The Macmillan Press Ltd. 1986

was taken as one which gave a 50% inhibition of colour formation compared to an HTLV-III antibody negative serum.

### Case summaries

*Case 1* A 26 year old female, presented in October 1983 with multiple nodular swelling on the head, face and body and marked weight loss. She had been unwell for 8 months prior to admission with general weakness and intermittent fever; no history of diarrhoea was given but her appetite was poor. She was married with 2 children and lived in South West Uganda. There was no history of promiscuous behaviour, drug abuse or blood transfusion.

Physical examination of the patient revealed moderate anaemia, gross general lymphadenopathy, moderate wasting with few nodules on the head and trunk (Figure 1) and none on the limbs. She had bilateral enlarged tonsils, a pleural effusion (R) side, liver enlargement 6 cm below the costal margin and moderate splenomegaly. She had a haemoglobin of $8.4\,g\,dl^{-1}$, WBC of $4.2\times10^{9}\,l^{-1}$ (N; 63% L; 34% E; 1%) and was HTLV III seropositive.

**Figure 1** Case number 1 showing nodules on the head.

A chest radiograph showed extensive infiltration of both lung fields. A pleural biopsy was positive for Kaposi's sarcoma (mixed cellularity). She was randomized to receive Adriamycin as a single agent and after 3 courses of chemotherapy she had a clinical reduction in tumour of 50%, including reduction in lymph node and skin nodule size. Her weight increased by 10 kg and the chest radiograph returned almost to normal.

Disease remained fairly stable, until July 1984 when her general condition had deteriorated, nodules on the head had regrown, new plaques had appeared on the medial aspect of the thigh with extensive infiltration to give a tourniquet-like effect. She was very dyspnoeic and the liver and spleen almost filled the abdomen. Chest radiograph showed the (R) pleura full of fluid and some fluid on the left. She developed progressive dyspnoeia and congestive cardiac failure and eventually died, despite chemotherapy, in November 1984. Post mortem examination showed Kaposi's sarcoma involving the tongue, tonsils, oesophagus, stomach and both large and small gut. There were also tumour nodules in the liver, spleen and intra-abdominal lymph nodes. In the chest tumour nodules were seen on the pleura (parietal), lungs and hilar lymph nodes. The diaphragm, pericardium and left ventricle were also found to have tumour deposits.

*Case 2* A 30 year old doctor was referred to Uganda Cancer Institute with a clinical diagnosis of disseminated Kaposi's sarcoma confirmed on histology as mixed cellularity. He presented at a small district hospital 6 months prior to admission with facial puffiness, generalised nodular skin lesions, general malaise, weight loss and abdominal pain associated with diarrhoea. He denied sexual promiscuity but had a steady sexual partner who developed lymphadenopathy 6 months later. A single child is apparently healthy. There was no history of blood transfusion, drug abuse or homosexuality.

On arrival the following findings were noted: he was very sick and had generalised oedema of the head and nodular skin lesions. He was wasted and dyspnoeic. There was a fungating purple mass in the oral cavity extending from the soft palate. The tongue was coated with oral thrush. There was generalised lymphadenopathy, bilateral pleural effusion, a palpable spleen and a diffuse mass in the right iliac fossa.

The cerebrospinal fluid was xanthochromic with a protein concentration of $>120\,mg\,dl^{-1}$ and no organisms were isolated. He had an Hb of $7.7\,g\,dl^{-1}$, WBC of $5.9\times10^{9}\,l^{-1}$, (L; 31%, N; 66%, E; 3%). The stool contained *Shistosoma mansoni*, and *Strongyloides stercolatis* and he was HTLV III seropositive. A repeat biopsy of the lymph node, pleura, oral and skin lesions all confirmed Kaposi's sarcoma (mixed cellularity).

The patient was given six courses of four drug combinations; Adriamycin, Vincristine, DTIC and Actinomycin D. He continued to deteriorate with minimal clinical response and died 6 months later with respiratory failure. Autopsy was not done.

*Case 3* A 44 year old depot manager from South West Uganda, married with 4 children, presented in March 1984 with general lymphadenopathy, 1 month history of diarrhoea (Novs *et al.*, 1974) and weight loss. There was no history of drug abuse, homosexual behaviour or blood transfusion.

Examination revealed general ill health, wasting with moderate anaemia, oral candidiasis, and enlarged tonsils bilaterally. He had hepatomegaly 3 cm below the sub-costal margin mid-clavicular line but no skin nodules or plaques.

Lymph node biopsy revealed mixed cellularity Kaposi's sarcoma. WBC; $3.5\times10^{9}\,l^{-1}$ (normal differential). He was seropositive for HTLV-III. Despite ADM as a single agent, he died with a massive pleural effusion and respiratory distress in May 1984. Autopsy was refused.

*Case 4* A 19 year old fisherman from South West Uganda married but with no children presented in November 1984 with general swelling of the head (Figure 2) prior to 1 month history of diarrhoea and intermittent fever. There was no history of drug abuse, homosexual behaviour or blood transfusion.

Physical examination was remarkable for the weight loss, nodules on the tongue and enlarged tonsils, nodules on the chest but none on the limbs; he had lymphadenopathy and hepatomegaly, chest was normal. WBC;

**Figure 2** Case number 4 showing general swelling of the head and nodules on the chest.

$4 \times 10^9 l^{-1}$ (L; 26%, N; 68%, E; 6%). HTLV III seropositive; histology of the lymph nodes showed monomorphic KS. He was given Adriamycin as a single agent with no response and died at home in February 1985.

From the pathology records a total of 194 cases of Kaposi sarcoma were registered between 1980–1984 in adult males of which 33 cases were generalized Kaposi sarcoma (17%). (Table I).

From 1980–1983 the percentage of cases which were generalized increased year by year until in 1983 it reached 31%. However in 1984 it fell back to 17.7%.

**Table I** Number of new cases of Kaposi's sarcoma in adult males

| | *1980* | *1981* | *1982* | *1983* | *1984* | *Total* |
|---|---|---|---|---|---|---|
| Endemic KS | 43 | 31 | 30 | 20 | 37 | 161 |
| Generalized KS | 3 (6.5%) | 3 (8.8%) | 10 (25%) | 9 (31%) | 8 (17.7%) | 33 |
| | 46 | 34 | 40 | 29 | 45 | 194 |

### Discussion

The common or peripheral form of Kaposi sarcoma has been classified as nodular and as locally aggressive by Templeton (1972). Taylor *et al.* (1971) further divided the locally aggressive form into infiltrative and florid type. This common or peripheral form is also described as endemic by Bayley (1984), but however described this disease presentation accounts for over 80% of cases in Uganda.

The major distinguishing features of aggressive disease as described by Bayley are: the absence of skin lesions, a poor response to conventional chemotherapy; its appearance in a younger population; disseminated disease usually involving lymph nodes; a poor prognosis and evidence of infection with HTLV-III.

From October 1983–December 1984 we saw four patients with generalized lymphadenopathy, oral lesions and visceral involvement or general swelling of the head. Cutaneous nodules/plaques on the limbs were not seen. The patients were young (average age 29.5 years; range 16–44 years) and the duration of illness prior to admission was relatively short (average 4 months; range 1–9 months); all the patients are now dead. One died within 2 months of admission with no response to chemotherapy, but the other three had an initial response. The average duration of survival from time of admission was 8 months (range 2–12 months). Histologically the disease was of the mixed cellularity type except in one case where monomorphic Kaposi's sarcoma was seen in a lymph node which is unusual in our experience and may indicate differences in the underlying pathology. All 4 patients had antibodies to HTLV-III. We conclude that the cases described here fit Bayley's description of aggressive KS.

Bayley also reported that she was seeing an increasing number of aggressive cases which prompted us to review the histological request forms at the Department of Pathology, Makerere University, between January 1st 1980 and December 1984. Overall 17% of the cases of KS in adult males during that period had generalized disease compared with only 3% in 1972 (Templeton, 1972) confirming an increased incidence of this form of KS. However the year by year increase seen from 1980–1983 was not maintained in 1984. It would appear then that generalized KS in the 4 Ugandan patients studied is the same disease as aggressive KS in Zambia. Overall the incidence of this form of KS has increased substantially since 1972.

The fact that all 4 patients in this study had generalised KS and were infected with HTLV-III suggests they were suffering from AIDS. Indeed as further evidence two of them had oral candidiasis, an early marker of severe immunosuppression (Roberts *et al.*, 1984). It is tempting to extrapolate from the present series of 4 patients to all cases of generalized disease seen in Uganda over the past 10 years and say that the generalized presentation is part of the AIDS spectrum. However this would be unjustified and probably wrong. It seems likely that KS can present as a generalized disease in response to a number of different factors and infection with a lymphotropic virus is only one of them. For example KS in older men tends to become more generalized even when the patient is not infected with HTLV-III and children with KS are another group where the disease is generalized but not necessarily associated with HTLV-III (unpublished observations). Nevertheless it is possible that a proportion of the patients with generalized KS seen in Kampala over the past 10 years, particularly those in their early adulthood, did have AIDS. The critical deciding factor is whether or not they were infected with HTLV-III. Although we may never know the answer to this question there is evidence that the virus was present in Uganda in 1972 (Saxinger *et al.*, 1985*a,b*). Sera collected in the West Nile District from healthy children showed a high prevalence (66%) of antibodies to HTLV-III (or a related virus) by ELIZA, later confirmed by western blotting. Furthermore recent serological data (Serwadda *et al.*, 1985) indicate that the virus is endemic; 10% of the healthy adult population who have no obvious risk factors are infected. Taken together these findings suggest that the origin of HTLV-III or a related virus in the Ugandan population is not recent.

If the virus has been present in Uganda since 1972 one has to ask why AIDS was not reported until this year (Serwadda *et al.*, 1985), given that disease surveillance has always been of a high standard. One possible explanation is that part of the population has immunity to the virus, perhaps those infected in early childhood, and that the current epidemic reflects escape by the virus to the non-immune population.

An equally plausible explanation is that the virus detected in 1972 is related to HTLV-III but is itself non-pathogenic. Such a virus might have been the predecessor from which the pathogenic AIDS virus arose by mutation. Distinguishing between the various alternatives is of vital importance to our understanding of the pathogenicity and eventual control of this virus.

We are indebted to Professor Bayley for reading the manuscript, to M. Roff for technical assistance and to J. Leese and G. Prentis for typing.

**References**

BAYLEY, A.C. (1984). Aggressive Kaposi's sarcoma in Zambia. *Lancet*, **i**, 1318.

BAYLEY, A.C., DOWNING, R.G. & CHEINGSONG-POPOV *et al.* (1985). HTLV-III serology distinguishes atypical and endemic Kaposi's sarcoma in Africa. *Lancet* **i**, 359.

CHEINGSONG-POPOV, R., WEISS, R.A., DALGLEISH, A.G., *et al.* (1984). Prevalence of antibody to HTLV-III in AIDS and patients at risk of AIDS in Britain. *Lancet*, **ii**, 477.

KYALWAZI, S.K. (1981). Kaposi's sarcoma: Clinical features. Experience in Uganda. *Antibiotics & Chemotherapy*, **29**, 59.

LEVY, J.A. & ZIEGLER, J.L. (1983). Acquired immune deficiency syndrome is an opportunistic infection and Kaposi's sarcoma results from secondary immune stimulation. *Lancet*, **ii**, 78.

LOTHE, F. (1963). Kaposi's sarcoma in Ugandan Africans. *Act. Pathol. Microbiol. Scand.*, **161**, Suppl. p. 71.

NOVS, H., KING, H., BANKS (1974). Kaposi's sarcoma presenting with diarrhoea and protein losing. *Gastroenterology*, **67**, 996.

OLWENY, C.L.M., TOYE, KATONGOLE-MBIDDE, E., LWANGA, S.K., OWOR, R., KYALWAZI, S.K., VOGEL, C.L. (1974). Treatment of Kaposi's sarcoma by combination of Act-D, Vincristine and Imidazole Carboxamide (DTIC). Results of randomized trials. *Int. J. Cancer*, **14**, 649.

OLWENY, C.L.M., KADDUMUKASA, A., ATINE, I., OWOR, R., MAGRATH, I., ZIEGLER, J.L. (1976). Childhood Kaposi's sarcoma. Clinical features and chemotherapy. *Br. J. Cancer*, **33**, 555.

OLWENY, C.L.M. (1981). Management of Kaposi's sarcoma. *Antibiotics & Chemotherapy*, **29**, 88.

ROBERTS, S.K., HARRIS, C.A. & SMALL, C.B. (1984). Oral candidiasis in high risk patients as the initial manifestation of AIDS. *N. Engl. J. Med.*, **311**, 354.

SAXINGER, W.C., LEVINE, P.H., DEAN, A.G *et al.* (1985*a*). Evidence for exposure to HTLV-III in Uganda before 1973. *Science*, **227**, 1036.

SAXINGER, C., LEVINE, P.M. & DEAN, A. *et al.* (1985*b*). Unique patterns of HTLV-III (AIDS-related) antigen recognition by sera from African children in Uganda (1972). *Cancer Res.*, **45**, 4624.

SERWADDA, D., SEWANKAMBO, N.K., CARSWELL, J.W. *et al.* (1985). SLIM disease: a new disease in Uganda and its association with HTLV-III infection. *Lancet*, **ii**, 849.

TAYLOR, J., TEMPLETON, A.C., VOGEL, C.L., ZIEGLER, J. & KYALWAZI, S.K. (1971). Kaposi sarcoma in Uganda; a clinicopathological study. *Int. J. Cancer*, **8**, 122.

TEMPLETON, A.G. (1972). Studies in Kaposi's sarcoma: Postmortem findings and disease patterns in woman. *Cancer*, **30**, 854.

VOGEL, C.L., PRIMACK, A., OWOR, R. & KYALWAZI, S.K. (1973). Effective treatment of Kaposi's sarcoma with Imidazole Carboxamide. *Cancer Chemotherapy Rep.* Part 1 **57**, 65.